# Is Cyanosis Exposure Associated with Exercise Capacity or Daily Physical Activity in Children with Complex Congenital Heart Disease: A Cross-Sectional Study

**DOI:** 10.3390/children13010003

**Published:** 2025-12-19

**Authors:** Chirag Karki, Tyler Kung, Joel Blanchard, Jane Lougheed, Vid Bijelić, Reza Belaghi, Patricia Longmuir

**Affiliations:** 1 Children’s Hospital of Eastern Ontario Research Institute, Ottawa, ON K1H 8L1, Canadajlougheed@cheo.on.ca (J.L.); vbijelic@cheo.on.ca (V.B.); rbelaghi@cheo.on.ca (R.B.); 2 Faculty of Health Sciences, University of Ottawa, Ottawa, ON K1N 6N5, Canada; 3 Faculty of Medicine, University of Ottawa, Ottawa, ON K1N 6N5, Canada

**Keywords:** sub-maximal exercise capacity, moderate-to-vigorous physical activity, tetralogy of Fallot, transposition of the great arteries, single ventricle

## Abstract

**Highlights:**

**What are the main findings?**

• Age and cardiopulmonary exercise test duration are the strongest predictors of daily physical activity behavior among children with congenital heart disease.
• Longer exposure to cyanosis negatively impacts the submaximal exercise capacity needed for an active lifestyle.

**What are the implications of the main findings?**

• Children with prolonged exposure to cyanosis are at increased risk for a sedentary lifestyle.
• Clinicians can estimate daily physical activity behavior from cardiopulmonary exercise test duration.

**Abstract:**

**Background/Objectives**: Children with complex congenital heart disease (CHD) often exhibit lower levels of physical activity, but whether chronic cyanosis exposure is associated with activity participation is unclear. This cross-sectional study investigated whether the duration of cyanosis prior to surgical correction was associated with submaximal or maximal exercise tolerance or daily habitual physical activity. **Methods**: Thirty-six children (10–17 years) with transposition of the great arteries (TGA), tetralogy of Fallot (TOF), or Fontan physiology were tested with cardiopulmonary exercise testing (Bruce treadmill protocol) and 7 days of accelerometry. Cyanosis duration from birth to surgery was calculated. **Results**: Only 17% of participants were meeting daily physical activity recommendations. Age and exercise time were the strongest predictors of activity behavior. Children with <2 months of cyanosis had peak VO_2_ comparable with normative data (105% predicted), while those with longer durations of exposure had reduced submaximal and peak capacity (*p* < 0.001). The direct effect of days exposed to cyanosis on daily physical activity was not statistically significant (*p* = 0.55) but the indirect effect via submaximal energy consumption was statistically significant (*p* = 0.05), suggesting that a longer duration of cyanosis exposure negatively impacted physical activity through its detrimental effect on submaximal exercise capacity. **Conclusions**: These findings suggest that children with prolonged cyanosis exposure are at higher risk for reduced submaximal exercise capacity, which has a negative impact on daily physical activity participation. Age and exercise test duration can accurately estimate daily physical activity behaviors. Interventions to support these patients require investigation due to their increased risk for morbidities associated with inactive lifestyles.

## 1. Introduction

Advances in medical and surgical treatment have greatly improved the survival of the 13 per 1000 children [1] born annually with congenital heart disease (CHD). Most of these children, even with complex CHD (e.g., functional single ventricle (Fontan), tetralogy of Fallot (TOF), transposition of the great arteries (TGA)), now survive to adolescence and adulthood [2]. Nevertheless, these children are often less active than their healthy peers [3], who in turn do not typically achieve the daily physical activity recommended for optimal health [4]. Physically active lifestyles are important for all children, including those with complex CHD, as they contribute to cardiovascular health, growth and development, academic learning and overall quality of life [5]. Being able to accurately understand the physical activity of children with CHD and the factors that influence their participation is important for their health and care.

Current clinical assessments rely primarily on maximal cardiopulmonary exercise tests [6]. However, voluntary activities typically involve submaximal effort, making submaximal assessment protocols more relevant to daily functioning and a child’s ability to engage in physical activity and peer play [7]. The limited research on submaximal exercise capacity among children with CHD does not clearly identify whether their cardiac disease creates constraints for submaximal exercise. Although reduced maximal exercise capacity is often used to guide physical activity recommendations for children with CHD, some studies suggest that submaximal exercise capacity is not limited in children with complex CHD [8] and, in fact, may even be enhanced [9].

One factor that has received little attention in relation to exercise capacity among children with complex CHD is the length of cyanosis exposure. While the hypoxia encountered during short-term training at altitude has positive benefits for skeletal muscle oxidative capacity and capillarization, sustained exposure to hypoxia has detrimental skeletal muscle effects [10]. Chronic or intermittent exposure to hypoxia among children with CHD can impair lung development [1] and cause decreased cognitive function, delayed motor development, and impaired attention and visual function [2]. All of these changes could impact children’s ability to develop the movement skills needed for active play. However, adaptive mechanisms among children with cyanotic CHD could potentially counteract these negative effects [11], so whether exposure to hypoxia due to an unrepaired CHD would lead to better or worse exercise performance in later life remains unclear. The purpose of this study was to compare exercise capacity and daily physical activity among children with complex CHD relative to their exposure to days of cyanosis. Specifically, we compared children with TGA, TOF, and Fontan physiology who typically have surgical interventions that decrease or eliminate the chronic hypoxic condition at approximately 1 week, 6 months or 2 years of age, respectively. By examining both exercise capacity and daily physical activity, this study sought to clarify how days of cyanosis exposure might influence the ability of children with complex CHD to achieve a healthy, active lifestyle. It was hypothesized that muscle oxidative capacity would increase with a short hypoxic exposure (e.g., TGA) and decrease with an increased duration of hypoxia [10]. Children experiencing only acute hypoxia (2 months or less [10]) would be expected to have an increased submaximal and peak exercise capacity relative to peers without hypoxia exposure (i.e., normative data). Chronic cyanosis would be expected to decrease submaximal and peak exercise capacity. Decreased exercise capacity would hypothetically lead to decreased physical activity participation in daily life.

## 2. Materials and Methods

This cross-sectional study was approved by the Research Ethics Board of the Children’s Hospital of Eastern Ontario (REB# 18 62X). Most participants provided written informed consent to study participation, with younger children providing assent in conjunction with parental written informed consent. Data collection occurred between 2018 and 2020.

### 2.1. Participants Describes the Methods Used to Recruit Participants

Children (male or female) were eligible for this study if they were 10 to 17 years of age and were followed in the pediatric cardiology clinic for one of three CHD diagnoses: transposition of the great arteries treated via arterial switch operation, biventricular repair of tetralogy of Fallot, or Fontan palliation of a functional single ventricle. All participants were cleared by their responsible cardiologist to participate in the study protocol and perform moderate-to-vigorous physical activity. Children were excluded if they had additional medical conditions or disabilities that would impact their ability to perform physical activity and/or the study protocol (i.e., cerebral palsy, Down syndrome), were taking cardiac medication that would alter the heart rate response to exercise, or had a cardiac intervention in the preceding six months.

### 2.2. Assessment Procedures

The cardiopulmonary exercise tests were performed in the cardiac clinic exercise testing laboratory. A certified exercise physiologist (TK) conducted each exercise test, in conjunction with an ECG technician. Pre-exercise testing instructions asked participants to refrain from: (i) eating for at least 2 h, (ii) caffeinated beverages for 2 h, (iii) alcoholic beverages for at least 6 h, (iv) smoking for 2 h and (v) performing strenuous exercise for 6 h prior to the assessment. Participants were also instructed to take any medication as prescribed. Before the test, each participant had their height (cm) and body mass (kg) measured. Each participant was equipped with a standard 12-lead ECG (CASE 8000; GE Medical Systems, Milwaukee, WI, USA), headgear to hold a fitted mouthpiece (for breath-by-breath assessment of aerobic capacity), nose clip and blood pressure cuff. Indirect calorimetry was measured using a Vmax Encore Metabolic Cart (Sensormedic, San Diego, CA, USA). Baseline measurements of oxygen consumption $(\dot{V}$O_2_), respiratory exchange ratio, blood pressure and heart rate were recorded for 10 min while the participant was seated until the resting heart rate was below 100 bpm, there were no ECG complications, and the child was acclimatized to breathing with the mouthpiece (RER < 0.85). To promote exercise equipment familiarization and confirm a quality ECG tracing during exercise, participants were asked to perform a 2 min warm-up stage (6% grade at 1.5 mph). After the warm-up stage, all participants then completed the standard Bruce treadmill protocol until voluntary or symptomatic exhaustion [12]. Although the Bruce protocol is a maximal exercise testing protocol, each stage of the Bruce protocol provides data regarding submaximal exercise capacity. After completion of the Bruce protocol, all participants completed a 2 min cool down at the same grade and speed as the initial warm-up stage. Heart rate (bpm), breath-by-breath measures of oxygen consumption and a standard 12-lead ECG were continually monitored through the warm-up, testing protocol and cool-down. Blood pressure and rating of perceived exertion (RPE 0-10 Borg Scale [13]) were collected during the last minute of each Bruce protocol stage.

Participants were provided with an omnidirectional Actical Z-series accelerometer (Philips Respironics, Murrysville, PA, USA) to measure their daily physical activity. Participants were asked to wear the accelerometer at the right iliac crest along the mid-axillary line of the body for 7 consecutive days. The participants were also asked to manually log the time they woke up, bedtime, the type of physical activities performed that day and whether or not the accelerometer was worn all day. Accelerometers were pre-set to record data in 15 s epochs.

A review of each participant’s medical chart provided demographic data (age, sex) and treatment history (surgical repair date, birth date, date of exercise assessment).

### 2.3. Outcome Variable Calculations

The outcome variables were calculated as follows:

• Average Daily Minutes of Moderate-to-Vigorous Physical Activity (MVPA): Standardized cutpoints for Actical accelerometers [14] were used to calculate the minutes each day spent in sedentary, light or moderate-to-vigorous physical activity. Valid accelerometer data required a minimum of 10 h of wear per day and a minimum of 4 days of data (including one weekend day). Non-wear time was defined by log sheets completed by the participants. The average minutes per day was calculated separately for weekdays and weekend days and the overall average value was calculated ([((5 × weekday) + (2 × weekend))/7]) as the variable for modeling of results.;
• Days exposed to cyanosis: Estimated as surgical repair date minus birthdate. While pre-surgical interventions (e.g., shunts) may have altered the degree of cyanosis exposure, surgical repair date was identified as the end of cyanosis exposure because the effects of cyanosis exposure are similar whether chronic or intermittent [2].;
• Days Since Cyanosis: Estimated as exercise test date minus surgical repair date;
• Exercise Duration: Total exercise time (minutes);
• Peak Heart Rate: The highest heart rate recorded during the Bruce protocol (beats/minute);
• Peak $\dot{V}$O_2_: Highest oxygen consumption ($\dot{V}$O_2_) value for a 30 s interval during the Bruce protocol relative to body weight (ml/kg/min);
• Stage 2 Heart Rate: Average heart rate over the last 30 s of Stage 2 of the Bruce protocol;
• Stage 2 $\dot{V}$O_2_: Average $\dot{V}$O_2_ over the last 30 s of Stage 2 of the Bruce protocol;
• Stage 2 RER: Average respiratory exchange ratio over the last 30 s of Stage 2 of the Bruce protocol;
• $\dot{V}$O_2_ @ VAT: Average $\dot{V}$O_2_ at the ventilatory anaerobic threshold.

### 2.4. Statistical Analyses

All statistical analyses were performed using SPSS version 28 (IBM Corp, Armonk, NY, USA, 2017) and R (version 4.2.0) including the lavaan and semPlot packages for structural equation modeling (SEM). Statistical significance was defined as a two-sided *p*-value less than 0.05. Outliers were identified as values exceeding 2 standard deviations from the mean and were reported transparently.

Descriptive statistics (means, standard deviations, medians, and interquartile ranges) were computed to summarize participant characteristics. Dependent variables were assessments of daily physical activity (sedentary, light, MVPA) and exercise capacity (exercise duration, peak h, peak $\dot{V}$O_2_, Stage 2 h/$\dot{V}$O_2_/RER, $\dot{V}$O_2_ at VAT). Potential independent model variables were sex, age, diagnosis, days exposed to cyanosis (categorized as low, moderate, high), height, weight, and resting heart rate. Recognizing that the degree of cyanosis will vary between children with the same CHD diagnosis, and within the same child over time, for this analysis it was assumed that such variability would be randomly distributed among each study group. Outcomes were visualized using scatterplots to highlight trends and relationships between variables. Pearson’s correlation coefficients were used to assess the bivariate relationships between variables, and variables with correlation coefficients greater than 0.8 were noted for potential multicollinearity. Key outcome measures included submaximal and peak cardiopulmonary exercise variables (e.g., $\dot{V}$O_2_ at VAT, Stage 2 $\dot{V}$O_2_, Peak $\dot{V}$O_2_), and accelerometer-derived estimates of daily physical activity (e.g., MVPA minutes/day). Group comparisons were evaluated using independent *t*-tests or one-way ANOVA as appropriate. Prior to multi-variable modeling, each independent model variable was checked for significant interactions using a univariate general linear model. Linear regression with backward variable selection was used to define the optimal model for each outcome. Eta-squared or partial eta-squared values were calculated to evaluate effect sizes (small > 0.01, medium > 0.06, large > 0.14 [15]).

To examine the potential mediating role of exercise capacity in the relationship between age, sex, and duration of cyanosis (as a continuous variable) with daily physical activity, structural equation modeling (SEM) was conducted. A conceptual path model was specified in which age, sex, and log-transformed days exposed to cyanosis predicted average daily moderate-to-vigorous physical activity (MVPA), both directly and indirectly through submaximal exercise capacity ($\dot{V}$O_2_ at ventilatory anaerobic threshold). The model was evaluated for goodness-of-fit using conventional indices (e.g., CFI, TLI, RMSEA).

## 3. Results

### 3.1. Participants Provides the Results from Participants

The convenience sample of 36 study participants are described in Table 1. All participants completed the cardiopulmonary exercise test. One 10-year-old male (Fontan) did not complete the accelerometry assessment. On average, males were taller than females and demonstrated longer exercise duration and higher peak exercise capacity. There were no significant differences in submaximal exercise measures or daily physical activity by diagnosis.

### 3.2. Cyanosis and Exercise Capacity

Children who experienced cyanosis for 2 months or less (11 TGA, 1 TOF) achieved a significantly higher (*p* < 0.001, 95% CI: 42, 55) peak oxygen consumption than children who experienced cyanosis for longer periods (95% CI: 30, 35). Children with short exposure to cyanosis achieved 105% ± 21% of predicted peak oxygen consumption for age and sex (95% CI: 91, 119). Children who experienced cyanosis for longer periods had significantly (*p* < 0.001) lower (95% CI: 70, 88) peak oxygen consumption relative to normative data. Children with limited cyanosis exposure (<2 months) achieved peak heart rates (95% CI: 180, 193 bpm) that were not significantly different (*p* = 0.18) from children with longer cyanosis exposure (95% CI: 169, 187) and both achieved approximately 90% of their predicted peak heart rate for age (95% CI: 88, 94 and 95% CI: 85, 94, respectively).

Measures of submaximal and peak exercise capacity differed significantly among those with low (less than 62 days; timeline that athletes use for high altitude training exercise benefits), medium (62 to 365 days) or high (more than 365 days; representing chronic exposure) exposure to cyanosis (Table 2). Aligned with our hypothesis, exercise capacity decreased with increasing length of cyanosis exposure. As noted in Table 1, participants with tetralogy of Fallot included one child who was repaired at 17 days of age and a second child not repaired until 1629 days. These children were thus included in the low and high cyanosis exposure groups, respectively.

### 3.3. Cyanosis and Physical Activity

There was no direct association between cyanosis exposure and daily physical activity (Table 3).

### 3.4. Predicting Physical Activity from Exercise Capacity

Age, sex, treadmill exercise duration and oxygen consumption during Stage 2 of the Bruce protocol were important factors in models of sedentary time and light physical activity (Table 4(a–c)). Age and exercise duration measures had the largest effect size for sedentary time and moderate-to-vigorous physical activity minutes, with only age having more than a small effect on light physical activity.

**Figure 1:**
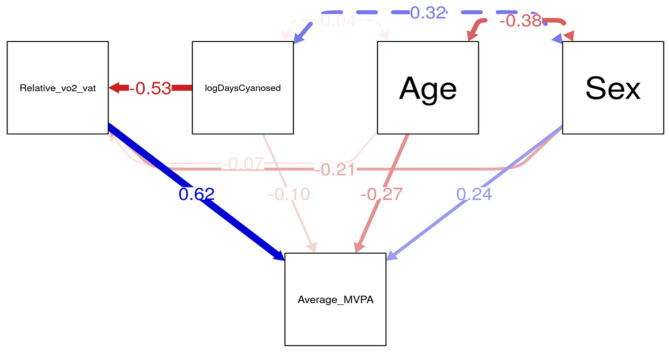
Structural Equation Modeling Path Diagram. Solid arrows denote direct effects and dashed arrows denote indirect effects. Blue and red arrows indicate positive and negative associations, respectively; arrow thickness reflects effect size. Values shown are standardized coefficients.

As shown in the SEM path diagram (Figure 1), longer cyanosis duration was significantly associated with reduced submaximal exercise capacity (standardized β = −0.53), as measured by the oxygen consumption at the ventilatory threshold ($\dot{V}$O_2_ @ VAT). As expected, submaximal exercise capacity was positively associated with MVPA (β = 0.62). Additionally, older age and male sex were negatively associated with MVPA (β = −0.27 and β = −0.24, respectively), with age also demonstrating a marginal indirect effect via reduced submaximal exercise capacity. These findings support a mediation effect of submaximal exercise capacity in the association between cyanosis duration and daily physical activity. The model accounted for a substantial proportion of the variance in MVPA (R^2^ ≈ 0.48), indicating good explanatory power.

To validate the mediation hypothesis, indirect effects were formally tested using bootstrapped confidence intervals (5000 iterations). The indirect pathway from days exposed to cyanosis to daily physical activity minutes (MVPA) via submaximal exercise capacity ($\dot{V}$O_2_ @ VAT) was statistically significant (*p* < 0.01), supporting the hypothesized mechanism whereby chronic cyanosis reduces exercise capacity, which in turn limits physical activity engagement. Model fit indices indicated an acceptable fit to the data (e.g., RMSEA < 0.05, CFI > 0.95), suggesting the proposed pathways reflect meaningful relationships among variables in this pediatric CHD cohort.

## 4. Discussion

This study evaluated the association between exposure to cyanosis and exercise capacity or daily physical activity among children with complex CHD. Children with only acute (2 months or less) exposure to cyanosis (11 TGA, 1 TOF) had significantly greater peak exercise capacity than children with longer exposure to cyanosis. Children with chronic exposure to cyanosis (TOF and Fontan) demonstrated peak exercise capacity that was significantly lower than values predicted for age/sex. Increasing length of cyanosis exposure was significantly related to decreasing measures of submaximal and peak exercise capacity.

The direct effect of days exposed to cyanosis on daily physical activity was not statistically significant (95% CI: −4.806 to 2.323, *p* = 0.55), indicating that when controlling for submaximal exercise capacity ($\dot{V}$O_2_ @ VAT), the direct pathway from cyanosis to physical activity was not distinguishable from zero. In contrast, the indirect effect via submaximal exercise capacity was statistically significant (95% CI: −6.953 to −0.362, *p* = 0.05), suggesting that a longer duration of cyanosis negatively impacted physical activity through its detrimental effect on submaximal exercise capacity. The total effect (Estimate = −4.389, 95% CI: −8.100 to −0.409, *p* = 0.02) was significant, implying that the overall association between cyanosis duration and reduced MVPA is meaningful when combining both direct and mediated pathways. Approximately 76.2% of the total effect was mediated through submaximal exercise capacity, highlighting the critical role of physiological fitness as a pathway through which chronic cyanosis may reduce physical activity levels in this pediatric cohort.

Athletes who train at altitude (i.e., in a hypoxic condition) experience positive effects on exercise capacity. Acute exposure to hypoxia increases hemoglobin content, free fatty acid substrate utilization and oxidative enzyme activity [4]. However, such athletes typically follow protocols that are limited to less than 2 months duration with the benefits dissipating after just a few weeks of returning to normoxic conditions [5]. Any acute benefits of a brief exposure to hypoxia in infancy would not be expected to be evident when exercise tests are completed after years of normal oxygen saturations.

The results of this study contribute to the growing body of literature demonstrating the importance of assessing submaximal exercise capacity and daily physical activity in populations with pediatric CHD. Historically, studies have mostly focused on measures of maximal exercise response and their relationship to adverse changes to cardiovascular function [6,7,8]. However, voluntary activity typically involves an intensity of only 60–80% of maximal capacity. At this level, aerobic energy sources are used and can sustain activity for a longer period of time. By contrast, anaerobic energy sources can sustain very high levels of exertion but only for a brief period (approximately 75 s [9]) before they decline. During active play, children’s heart rates are approximately 150 beats/minute with oxygen consumption rates of 25–30 mL/kg/min [10] indicating that submaximal exercise capacity would be more indicative of children’s normal play and physical activity. In this study, children with only acute cyanosis exposure continued to rely primarily on aerobic energy systems during Stage 2 of the Bruce protocol. However, children with chronic cyanosis exposure were relying on both aerobic and anaerobic energy systems to complete the same workload. The need to engage anaerobic energy systems at a lower workload would therefore reduce total exercise duration, and more significantly limit their engagement in active childhood play. While it is recognized that there are a large variety of biological, psychological, social and environmental factors associated with the physical activity participation of all children [11], children with complex CHD face the additional risks associated with cyanosis exposure (e.g., delayed motor development, decreased cognitive function, limited attention). Examining the submaximal exercise capacity of children with CHD in light of their exposure to cyanosis may provide more accurate guidance regarding their capacity for a healthy, active lifestyle with peers.

Studies have found that while a small proportion of children with CHD meet daily physical activity recommendations [12], children with complex CHD are often less active than healthy children [13,14]. In our study, only 6 of 35 participants (17%) were performing the recommended 60 min of moderate-to-vigorous intensity activity recommended per day [15] and there was no direct relationship between cyanosis exposure and daily physical activity habits. These results support the importance of the multifaceted barriers to physical activity participation that have been identified in individuals with CHD, beginning in childhood and persisting into adulthood [16]. The inter-individual variability highlights the need for individualized, developmentally appropriate strategies to encourage long-term physical activity involvement in this population [14,16]. The potential impact of the duration of cyanosis on children’s physiological capacity for physical activity, and how that might interact with other barriers or facilitators to physical activity requires further investigation.

A highly novel contribution of this study is the use of “days exposed to cyanosis” as both a categorical and a continuous variable to predict the performance of submaximal exercise and daily physical activity. Unlike categorical groupings based on diagnosis alone, this variable allowed us to examine the physiological consequences of chronic exposure to cyanosis with greater nuance since our study sample included two children with the same CHD diagnosis (tetralogy of Fallot) whose exposure to cyanosis was similar to children with other diagnoses (transposition of the great arteries and single ventricle). Our findings show that longer cyanosis duration is associated with reduced submaximal exercise capacity, which in turn is associated with reduced daily physical activity. Aligned with acute hypoxia training effects, patients with very limited cyanosis exposure (<2 months) had comparable or slightly higher exercise capacity than predicted for children of similar age and sex. This study suggests the need to further evaluate the impact of cyanosis exposure on the capacity of children with complex CHD to achieve the physically active lifestyles associated with optimal health.

A primary strength of the study was the even distribution of participants among the three diagnostic groups and the use of both sexes, which increases generalizability across subtypes of pediatric CHD. Additionally, the use of objective accelerometer assessments of physical activity enhances the validity of these measurements [17]. Participants included those with a wide range of cyanosis duration from a few days to several years, including two TOF patients with 17 and 1629 days of exposure. This variability allowed more refined examination of cyanosis as a predictor variable.

A key limitation of this study was the relatively small sample size (N = 36), which restricted the degrees of freedom available for model estimation and constrained the complexity of the structural equation modeling. The proposed mediation model was just-identified, meaning that while all pathways were estimable, formal model fit indices could not be interpreted due to the model’s saturated structure. Nevertheless, bootstrap methods were employed to estimate the indirect effects and assess the robustness of the mediation hypothesis. The findings provide preliminary support for the hypothesis that prolonged cyanosis adversely affects submaximal exercise capacity, which in turn limits daily physical activity. However, the results should be interpreted with caution, and future studies with larger samples are necessary to confirm the mediation effect, evaluate model fit more rigorously, and explore additional mediators or moderators (e.g., socio-environmental factors or cardiac function indices).

One participant did not complete the accelerometer assessment and the ability of participants to provide a maximal effort during the treadmill testing varied. Two (6%) participants did not transition to anaerobic energy systems (i.e., RER > 1.0) even at peak effort. Therefore, these results reflect the peak voluntary effort of participants and not necessarily their physiological maximum capacity. Given this study’s focus on submaximal exercise, hemoglobin concentration was not measured at the time of exercise testing. Although changes in hemoglobin concentration result in changes to maximal exercise capacity, during submaximal exercise such changes can be offset by an increase in muscle blood flow [18]. It is recognized that a wide number of variables impact exercise capacity (e.g., cardiac function, residual lesions, oxygen saturation) and physical activity participation (e.g., age, sex, social support, environmental opportunities). Analyzing the impact of these variables in relation to cyanosis exposure would require a substantially larger study sample. The current analyses are based on the assumption that these other variables would be randomly distributed among the study participants. The interpretation of these results relied on published normative data rather than a contemporary control assessed with the same protocol. Families who consented to participate may represent more active or health-conscious demographics compared to the broader CHD population.

## 5. Conclusions

The exposure to cyanosis that occurs among children with complex CHD prior to surgical correction may be an important influence on their future exercise capacity and daily physical activity. Longer-term exposure to cyanotic conditions was associated with decreasing capacity for submaximal exercise and decreased participation in daily physical activity. These findings align with data from healthy adults exposed to hypoxic conditions at high altitudes. Participant age and total exercise duration during the Bruce treadmill protocol were the most important factors directly associated with daily physical activity habits. These results suggest that although children may vary in their physiological responses to exercise, exposure to cyanosis may be an important factor in that variability, and that it is how children adapt to these physiological differences to extend their exercise duration that is most relevant to their capacity for a healthy, active lifestyle. Individually tailored interventions to encourage physical activity may be able to bridge the gap between lifestyle activity and physiological capacity. Long term studies should continue to explore the long-term impact of early cyanosis on cardiopulmonary adaptation and establish how environmental and behavioral factors modulate physiological capacity in this at-risk population.

## Figures and Tables

**Table 1:** Description of Study Participants.

Variable	All Participants	Males	Females
Number of Participants	36	25	11
ge (years)	13.7 ± 2.4	14.2 ± 2.2	12.6 ± 2.4
Height (cm) *	158.6 ± 15.4	162.1 ± 15.1	150.5 ± 13.4
Weight (kg)	54.9 ± 19.1	57.3 ± 19.9	49.5 ± 16.9
Resting Heart Rate (beats/mins)	86 ± 15	87 ± 15	83 ± 18
Exercise Duration (mins) *	8.8 ± 2.4	9.5 ± 2.5	7.4 ± 1.3
Peak Heart Rate (beats/min)	181 ± 18	184 ± 18	175 ± 18
Peak Oxygen Consumption (ml/kg/min) *	37.6 ± 10.4	39.9 ± 10.9	32.5 ± 7.2
Stage 1 Heart Rate (beats/min)	114 ± 18	112 ± 19	118 ± 16
Stage 1 Oxygen Consumption (ml/kg/min)	18.3 ± 2.3	18.6 ± 1.9	17.7 ± 3.1
Stage 2 Heart Rate (beats/min)	146 ± 25	144 ± 28	149 ± 20
Stage 2 Oxygen Consumption (ml/kg/min)	25.7 ± 3.7	26.2 ± 3.2	24.6 ± 4.5
Sedentary Time (mins/day)	772 ± 135	776 ± 94	762 ± 203
Light Active Time (mins/day)	124 ± 42	117 ± 43	140 ± 39
Moderate-to-Vigorous Active Time (mins/day)	40 ± 22	38 ± 23	44 ± 19
Meeting Daily Activity Guideline **	Yes = 6	Yes = 3	Yes = 3
	No = 29	No = 21	No = 8
	Missing = 1	Missing = 1	Missing = 0
CHD Diagnosis			
TGA	11	10	1
TOF	13	7	6
Fontan	12	8	4
Days exposed to cyanosis			
TGA	9 ± 3	78 ± 2	14
TOF ***	179 ± 68	179 ± 76	180 ± 68
Fontan	1248 ± 312	1171 ± 183	1401 ± 481
Days Since Cyanosis			
TGA	5268 ± 879	5401 ± 802	3940
TOF	5188 ± 856	5551 ± 752	4886 ± 880
Fontan	3787 ± 840	4163 ± 763	3036 ± 316

* Variable is significantly different between males and females (*p* < 0.05). ** Meeting the guideline was defined as achieving a daily average of at least 60 min of moderate-to-vigorous physical activity as per the World Health Organization recommendations [3]. *** One child had a biventricular repair of tetralogy of Fallot (TOF) at only 17 days of age. A second child did not have surgical TOF repair until 1629 days (4.5 years of age). These two participants are not included in the calculations of days exposed to cyanosis for males, females or overall reported in this table.

**Table 2:** Decreased Submaximal and Peak Exercise Capacity with Increased Cyanosis Exposure.

Exercise Measure ^1^	Low Cyanosis (n = 12)	Medium Cyanosis (n = 11)	High Cyanosis (n = 13)	Significance
Exercise duration	10.8 ± 2.7	8.0 ± 1.7	7.7 ± 1.4	0.001 ^2^
Stage 2 $\dot{V}$O_2_	28.4 ± 3.6	24.4 ± 3.3	24.6 ± 3.1	0.02 ^2^
Stage 2 heart rate	133 ± 21	154 ± 22	150 ± 28	0.08
Stage 2 RER	0.92 ± 0.07	0.99 ± 0.04	1.0 ± 0.07	0.02 ^3^
Peak $\dot{V}$O_2_	48.1 ± 9.3	33.4 ± 7.3	31.5 ± 5.2	<0.001 ^4^
% predicted $\dot{V}$O_2_	105 ± 21	88 ± 25	71 ± 13	<0.001 ^2^
Peak heart rate	187 ± 10	181 ± 20	175 ± 22	0.28
$\dot{V}$O_2_ @ HR 130	27.7 ± 5.6	20.5 ± 5.6	22.3 ± 5.0	0.008 ^1^
$\dot{V}$O_2_ @ VAT	35.2 ± 9.2	25.3 ± 3.5	23.3 ± 7.3	<0.001 ^3^

^1^$\;\dot{V}$O_2_ = oxygen consumption relative to body weight (ml/kg/min); RER = respiratory exchange ratio; % predicted = percentage of oxygen consumption achieved for predicted value for age and sex; @ hr 130 = at a heart rate of 130 bpm; @ VAT = at ventilatory anaerobic threshold; Low Cyanosis = <62 days; Medium Cyanosis = 62–365 days; High Cyanosis = >365 days. ^2^ Low exposure group significantly different from medium and high exposure groups. ^3^ Low exposure group significantly different from high exposure group. ^4^ Low and medium exposure groups significantly different from high exposure group.

**Table 3:** Association between Cyanosis Exposure and Daily Physical Activity.

Activity	Low Cyanosis	Medium Cyanosis	High Cyanosis	Significance
Sedentary Minutes	733 ± 97	832 ± 172	755 ± 121	0.19
Light Minutes	138 ± 46	115 ± 45	119 ± 37	0.41
MVPA ^1^ Minutes	50 ± 26	31 ± 17	39 ± 18	0.10
Percent Sedentary	80 ± 7	85 ± 6	82 ± 7	0.15
Percent Light	15 ± 5	12 ± 5	13 ± 5	0.32
Percent MVPA	5.5 ± 2.9	3.2 ± 1.6	4.3 ± 2.2	0.07 ^2^

^1^ MVPA = moderate-to-vigorous physical activity. ^2^ Difference between low and medium exposure groups was significant (*p* = 0.05).

**Table 4:** Model of (a) Increased Percentage of Daily Sedentary Time, (b) Increased Daily Light Physical Activity, (c) Increased Daily Moderate-to-Vigorous Physical Activity Using Cardiopulmonary Exercise Parameters.

(a)				
Model Variable ^1^	Beta ± SE	Beta 95% CI	*p* Value	Partial Eta^2^
Intercept	70.9 ± 9.5	51.4, 90.5	<0.001	0.66
Older Age	1.9 ± 0.4	1.1, 2.8	<0.001	0.43
Shorter Exercise Duration	1.1 ± 0.4	0.2, 1.9	<0.001	0.20
Male Sex	3.0 ± 2.0	−1.1, 7.2	0.15	0.07
Lower $\dot{V}$O_2_ Bruce Stage 2	0.2 ± 0.3	−0.3, 0.7	0.47	0.02
**(b)**				
**Model Variable ^2^**	**Beta ± SE**	**Beta 95% CI**	***p*** **Value**	**Partial Eta^2^**
Intercept	21.8 ±7.2	7.1, 36.5	0.003	0.28
Younger Age	1.3 ± 0.3	0.7, 2.0	<0.001	0.38
Female Sex	2.0 ± 1.5	−1.1, 5.1	0.20	0.06
Longer Exercise Duration	0.4 ± 0.3	−0.2, 1.1	0.17	0.07
Higher $\dot{V}$O_2_ Bruce Stage 2	0.2 ± 0.2	−0.2, 0.6	0.29	0.04
**(c)**				
**Model Variable ^3^**	**Beta ± SE**	**Beta 95% CI**	***p*** **Value**	**Partial Eta^2^**
Intercept	5.3 ± 2.4	0.5, 10.1	0.03	0.15
Younger Age	0.5 ± 0.2	0.2, 0.8	0.002	0.28
Female Sex	1.3 ± 0.8	−0.2, 2.8	0.09	0.10
Longer Exercise Duration	0.05 ± 0.2	0.2, 0.9	0.005	0.24
Fontan Diagnosis	1.3 ± 0.7	−0.2, 2.8	0.08	0.10
TGA Diagnosis	1.4 ± 0.9	−0.5, 3.3	0.14	0.07

^1^ Model R^2^ = 0.48. ^2^ Model R^2^ = 0.44. ^3^ Model R^2^ = 0.47.

## Data Availability

Data are available upon request from the corresponding author for approved study protocols. A data sharing agreement will be required.

## Abbreviations

The following abbreviations are used in this manuscript:

CHD	Congenital heart disease
TGA	Transposition of the great arteries
TOF	Tetralogy of Fallot
Fontan	Functional single ventricle
ECG	Electrocardiography
$\dot{V}$O_2_	Rate of oxygen consumption
RER	Respiratory exchange ratio
VAT	Ventilatory anaerobic threshold
MVPA	Moderate-to-vigorous physical activity
